# Incidental findings on routine preoperative noncontrast chest computed tomography and chest radiography prior to cardiac surgery in the multicenter randomized controlled CRICKET study

**DOI:** 10.1007/s00330-022-09001-0

**Published:** 2022-07-19

**Authors:** Wiebe G. Knol, Annemarie M. den Harder, Linda M. de Heer, Kálmán Benke, Pál Maurovich-Horvat, Tim Leiner, Béla Merkely, Gabriel P. Krestin, Ad J.J.C. Bogers, Ricardo P.J. Budde

**Affiliations:** 1 Department of Cardiothoracic Surgery, Erasmus MC, Rotterdam, The Netherlands; 2 Department of Radiology & Nuclear Medicine, Erasmus MC, PO BOX 2040, ND-547, 3000 CA Rotterdam, The Netherlands; 3 Department of Radiology, University Medical Center Utrecht and Utrecht University, Utrecht, The Netherlands; 4 Department of Cardiothoracic Surgery, University Medical Center Utrecht and Utrecht University, Utrecht, The Netherlands; 5 Department of Cardiovascular Surgery, Heart and Vascular Center, Semmelweis University, Budapest, Hungary; 6 Department of Cardiology, Heart and Vascular Center, Semmelweis University, Budapest, Hungary; 7 Department of Radiology, Medical Imaging Center, Semmelweis University, Budapest, Hungary

**Keywords:** Tomography, X-ray computed, Cardiac surgical procedures, Preoperative care

## Abstract

**Objective:**

To describe the prevalence and consequences of incidental findings when implementing routine noncontrast CT prior to cardiac surgery.

**Methods:**

In the multicenter randomized controlled CRICKET study, 862 adult patients scheduled for cardiac surgery were randomized 1:1 to undergo standard of care (SoC), which included a chest-radiograph, or an additional preoperative noncontrast chest CT-scan (SoC+CT). In this subanalysis, all incidental findings detected on the chest radiograph and CT-scan were analyzed. The influence of smoking status on incidental findings was also evaluated, adjusting for sex, age, and group allocation.

**Results:**

Incidental findings were observed in 11.4% (*n* = 49) of patients in the SoC+CT group and in 3.7% (*n* = 16) of patients in the SoC-group (*p* < 0.001). The largest difference was observed in findings requiring follow-up (SoC+CT 7.7% (*n* = 33) vs SoC 2.3% (*n* = 10), *p* < 0.001). Clinically relevant findings changing the surgical approach or requiring specific treatment were observed in 10 patients (1.2%, SoC+CT: 1.6% SoC: 0.7%), including lung cancer in 0.5% of patients (*n* = 4) and aortic dilatation requiring replacement in 0.2% of patients (*n* = 2). Incidental findings were more frequent in patients who stopped smoking (OR 1.91, 1.03–3.63) or who actively smoked (OR 3.91, 1.85–8.23).

**Conclusions:**

Routine CT-screening increases the rate of incidental findings, mainly by identifying more pulmonary findings requiring follow-up. Incidental findings are more prevalent in patients with a history of smoking, and preoperative CT might increase the yield of identifying lung cancer in these patients. Incidental findings, but not specifically the use of routine CT, are associated with delay of surgery.

**Key Points:**

*• Clinically relevant incidental findings are identified more often after a routine preoperative CT-scan, when compared to a standard of care workup, with some findings changing patient management.*

*• Patients with a history of smoking have a higher rate of incidental findings and a lung cancer rate comparable to that of lung cancer screening trials.*

*• We observed no clear delay in the time to surgery when adding routine CT screening.*

**Supplementary Information:**

The online version contains supplementary material available at 10.1007/s00330-022-09001-0.

## Introduction

In patients scheduled for cardiac surgery, a preoperative CT scan is frequently performed prior to minimally invasive surgery, reoperations, aortic surgery and in patients with complex cardiac anatomy, to rule out coronary artery disease, or to screen for aortic atherosclerosis [1]. Screening for aortic calcification was also the primary aim of the multicenter randomized controlled CRICKET study. This study evaluated the effect of adding routine preoperative CT on top of standard of care, including only a preoperative chest radiograph (CXR), on the rate of change in surgical approach and perioperative stroke [2]. Several other studies also describe increased use of CT in the preoperative workup of patients scheduled for cardiac surgery [3–5]. As a consequence, it was noted that the prevalence of incidental findings is high in chest CT [6]. This is influenced by smoking history, with more noncardiac incidental findings in patients with active or former smoking [7]. Although some studies have reported on incidental findings with preoperative CT, these studies evaluated selected patient populations rather than routine use of CT [8–10]. Additionally, no previous studies have compared the prevalence of incidental findings on preoperative CT to the usual preoperative workup, or evaluated the effect of incidental findings on the timing of surgery. Therefore, this secondary analysis of the CRICKET study aimed to describe the effect of incidental findings when implementing routine noncontrast chest CT prior to cardiac surgery. In addition, we evaluated the influence of smoking on the prevalence of incidental findings in these patients.

## Methods

The study was approved by the local medical ethics committee (3-692/M), registered at clinicaltrials.gov (NCT02173470) and all patients provided written informed consent. Between September 2014 and October 2019, 862 patients scheduled for cardiac surgery were included in the CRICKET study. Patients with a recent CT-scan (≤3 months) were not included. The primary aim was to evaluate the effect of screening for aortic calcifications with routine preoperative CT on perioperative stroke. Patients were randomized 1:1 to undergo preoperative noncontrast chest CT in addition to standard of care (SoC+CT) or standard of care (SoC) alone. SoC in our practice routinely included a CXR, on top of the standard preoperative imaging comprised by an echocardiography and a coronary angiography. The inclusion and exclusion criteria, as well as the patient workflow, have been described previously [2].

### Imaging evaluation

A noncontrast CT-scan of the chest was acquired, ranging at least from the proximal aortic arch branches cranially to the entire heart caudally. Both CXR and the CT-scans were evaluated as part of the usual clinical care by a consultant radiologist. For the purpose of this analysis, incidental findings were defined as all findings on the CXR and CT-scan that required preoperative workup or follow-up, except for findings related to aortic calcification, since this was the primary objective of the trial. Subsequently, the findings were classified as “direct” when direct action was required, such as preoperative consultation of another specialty, additional invasive or non-invasive evaluation, and postponement or cancellation of the surgery. The finding was classified as “follow-up” if no direct action was required, but follow-up or postoperative consultation of another specialty was indicated. An exception was made for findings on CXR that initially required confirmation by a CT-scan, to prevent false inflation of direct findings in the SoC-group. When the CT-scan ruled out a clinically relevant finding, it was scored as follow-up regardless of the timing of the CT scan. If the suspected findings were confirmed, they were scored as “direct” or “follow-up,” depending on the subsequent management. For all findings, a distinction was made between pulmonary and non-pulmonary findings.

### Outcomes

The primary outcome for this subanalysis was the prevalence of all incidental findings in the SoC+CT group compared to the SoC group. Secondary outcomes were the incidental findings subcategorized in pulmonary and non-pulmonary findings and those requiring direct action or follow-up, diagnosis and management of all incidental findings in both groups, and delay between inclusion and surgery. As data were available until discharge after surgery, the diagnosis at the time of discharge was used. To further evaluate the clinical consequences of the findings, the subsequent management was classified into the following classes: “CT” for incidental findings on CXR that were ruled out with CT; “non-invasive evaluation” for any additional examination including follow-up and consultation of other specialties; “invasive evaluation” if invasive diagnostic procedures were used, including concomitant diagnostic procedures performed during the index cardiac surgical procedure; “changed approach” if the finding led to alteration, postponement, or cancellation of surgery; “treatment” if the finding led to a treatment specifically for that particular finding. The management was scored in the abovementioned order with only one classification per patient. For example, patients with “treatment” related to the incidental finding thus were not categorized as receiving a (non-)invasive evaluation, despite possibly undergoing additional evaluation prior to treatment.

Additionally, the effects of the additional preoperative CT and of a history of smoking on the rate of incidental findings were evaluated. This effect was adjusted for age, sex, and group allocation. Finally, the delay caused by incidental findings was evaluated by comparing the period between inclusion and surgery between the SoC+CT and SoC groups. The different patient recruitment strategies were accounted for by stratifying on center. Patients enrolled in the Semmelweis University were excluded for this analysis, because the timing of inclusion and study CT-scan varied between preoperative visit and preoperative admission, hampering the interpretability of the evaluation. The timing of the study CT differed between the remaining centers: at the Erasmus Medical Center, the study CT was acquired directly during the visit, but at the University Medical Center Utrecht, the study CT was acquired a day before surgery. One patient undergoing surgery a year after inclusion partially at request of the patient, was excluded from this analysis.

### Statistical analysis

Continuous data were presented as a mean with standard deviation when normally distributed, or as a median with the interquartiles otherwise. For the primary endpoint, confidence intervals were calculated using the Clopper-Pearson method. Categorical data were presented as counts and percentages. The rate of incidental findings and its different classes were compared between both groups using Fishers’ exact test. To evaluate the influence of smoking status, a binomial logistic regression model was fitted with the rate of any incidental finding as dependent variable. Sex, age, group allocation, and smoking status (never, stopped, or active smoker) were used as predictors. Subsequently, a more extensive model was fitted, including a polynomial term for age, to assess any non-linear effect of age. This model was compared to the linear model using the likelihood ratio test, keeping the more extensive model when it performed significantly better. Patients with missing values for smoking status were excluded from this analysis, assuming this status to be missing at random. Potential outliers and influential points were assessed using Cook’s distance. To compare the time between CT-scan and surgery, a Cox regression model was used, stratifying for center. Group allocation and presence of incidental findings were evaluated as univariate predictors and evaluated in a multivariate model including an interaction term between the two. The proportional hazards assumption was checked by transforming the Kaplan-Meier estimate in the log-log scale. Schoenfeld residuals were used to visually explore both the proportional hazards assumption and the time invariance of all covariates. No further model selection was applied. Patients who did not undergo surgery were excluded from this analysis. Statistical analyses were performed using SPSS version 25 (SPSS Inc) and R (version 3.6.1; R Foundation for Statistical Computing).

## Results

The final study sample consisted of 862 patients, of which 429 were randomized to the SoC+CT group and 433 to the SoC-group. Baseline characteristics are shown in Table 1. The prevalence of incidental findings and the subcategories are shown in Table 2. Any incidental finding was observed in 11.4% (*n* = 49) of patients in the SoC+CT group and in 3.7% (*n* = 16) of patients in the SoC-group (*p* < 0.001). When subcategorizing these findings, “direct” action was required in 3.7% (*n* = 16) of the SoC+CT group vs 1.4% (*n* = 6) in the SoC-group (*p* = 0.032), while the difference was even more pronounced with findings requiring “follow-up” (SoC+CT 7.7% (*n* = 33) vs SoC 2.3% (*n* = 10), *p* < 0.001).

**Table 1:** Baseline characteristics

Characteristic	SoC group (*n *= 433)	SoC+CT group (*n *= 429)
Age (years, mean ± SD; range)	66 ± 11; (23–88)	66 ± 10; (26–98)
Sex		
Male	74.1% (321/433)	69.9% (300/429)
Female	25.9% (112/433)	30.1% (129/429)
Diabetes		
Oral medication	15.5% (67/433)	11.9% (51/429)
Insulin dependent	7.6% (33/433)	6.8% (29/429)
Hypertension	65.3% (282/432)	68.3% (291/426)
Smoking		
Currently	12.3% (52/424)	13.7% (57/417)
Stopped smoking	41.7% (177/424)	37.6% (157/417)
COPD	10.6% (46/433)	13.5% (58/429)
Chronic kidney disease	15.9% (69/433)	17.0% (73/429)
Peripheral obstructive arterial disease	6.7% (29/433)	5.6% (24/429)
Atrial fibrillation	16.4% (71/433)	17.8% (76/429)
EuroScore II (in %, median [Q1–Q3])	1.31% [0.85–2.29]	1.35% [0.88–2.27]
Prior myocardial infarction	19.0% (82/432)	16.3% (70/429)

No significant differences were present between groups after randomization. Proportions are given as % (*x*/*n*), with *x* indicating the number of patients with the variable of interest, and *n* the number of patients with available data

*COPD*, chronic obstructive pulmonary disease

EuroScore II, a validated score predicting the risk of in-hospital mortality [18]

**Table 2:** Incidental findings per group, including the subcategories

	SoC (*n* = 433)	SoC+CT (*n* = 429)	*p*-value
Incidental finding	3.7% (16) 95% CI [2.1–6.0]	11.4% (49) 95% CI [8.4–14.6]	< 0.001
Direct	1.4% (6)	3.7% (16)	0.032
Pulmonary	0.9% (4)	1.9% (8)	0.26
Non-pulmonary	0.5% (2)	1.9% (8)	0.063
Follow-up	2.3% (10)	7.7% (33)	< 0.001
Pulmonary	1.6% (7)	6.5% (28)	< 0.001
Non-pulmonary	0.7% (3)	1.2% (5)	0.50

The incidental findings per group were categorized as those requiring “direct” action and those that could be managed during follow-up. The latter included all findings that were deemed irrelevant after confirmatory CT-scan in the SoC group

**Fig. 1:**
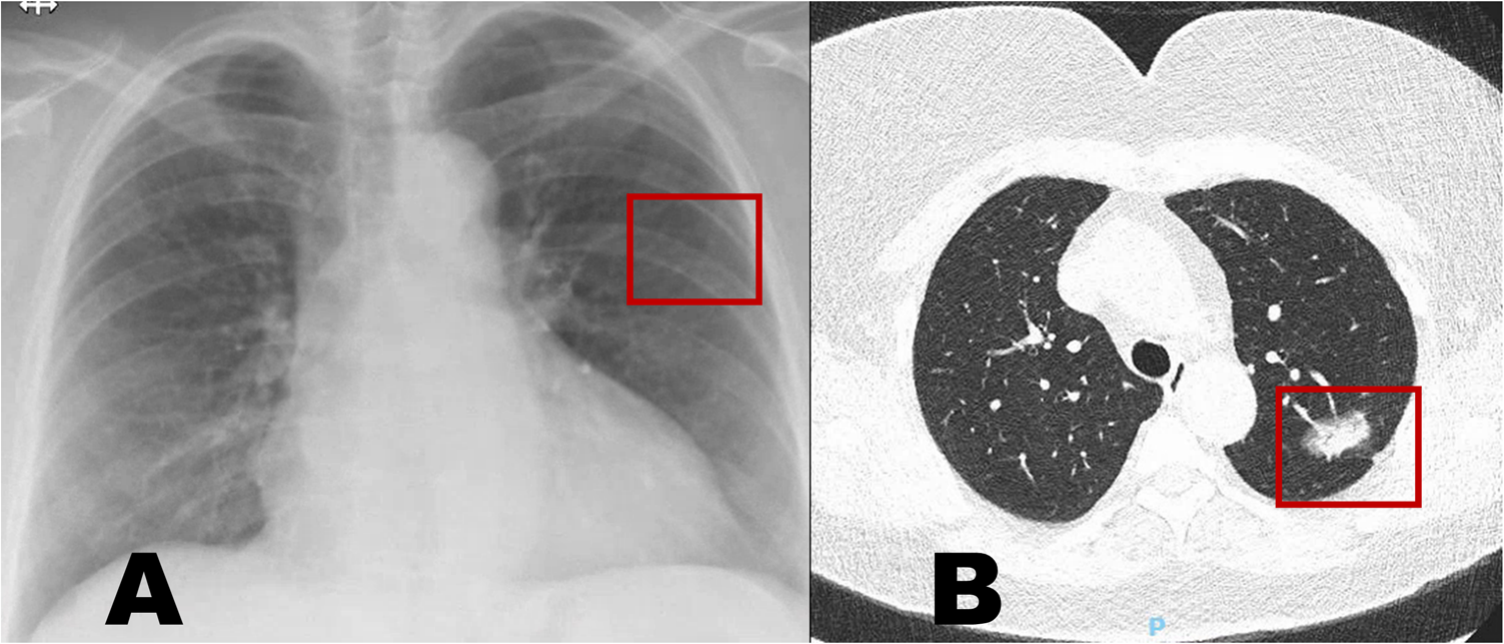
Sample images of a patient with lung cancer. **A** The chest radiograph from a patient in the SoC+CT group, where no clear abnormality was identified on CXR (red rectangle highlighting the region of the mass), but a density was clearly visible on the CT-images (**B**). Further workup of the clearly defined density on CT led to the diagnosis of a lung cancer

The diagnosis and management of all incidental findings in both groups are shown in Table S2 and Table S3. Both in the SoC group and the SoC+CT group, treatment was necessary for three patients, of whom two patients in each group were diagnosed with lung cancer. Sample images of patients with a lung cancer from the SoC+CT-group are shown in Fig. 1. In the SoC group, the planned surgery was not changed in any additional patients, while in the in the SoC + CT group, the planned surgery was changed in four patients. In both groups, an invasive workup was needed to rule out significant pathology in three patients, with two biopsies and one punction in both groups. In six patients in the SoC group, the incidental finding on CXR was not confirmed on CT, obviating further workup.

**Fig. 2:**
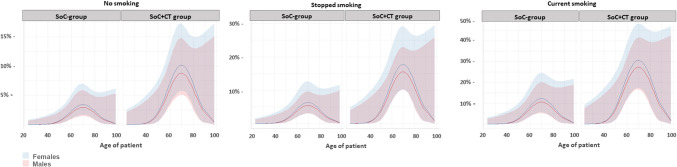
Effect of Age on rate of incidental findings adjusted for sex, randomization, and smoking status. The graphs show the probability of an incidental finding predicted by the logistic regression model, in patients from both groups, for males (red line, red area = 95% confidence interval) and females (blue line, blue area = 95% confidence interval). Note that the probability on the *y*-axis differs between the graphs for non-smokers, former smokers, and active smokers

The rate of incidental findings was more prevalent in older patients. In our logistic regression model, we observed a non-linear effect with a peak around 70 years of age, as shown in Fig. 2. Addition of a routine CT scan significantly increased the rate of incidental findings (Table 3). A history of smoking also increased this rate, with a higher odds ratio in active smokers (3.91) than in patients who stopped smoking (1.91). Of the four patients with lung cancer, two actively smoked (1.8%, 2 out of 109 patients) and two had a history of smoking (0.6%, 2 out of 333 patients).

**Table 3:** Influence of randomization and smoking history on rate of incidental findings

	Odds ratio	95% CI	*p*-value
*Intercept*	7.07 * 10^−11^	2.02*10^−19^–1.70*10^−4^	0.008
Age	1.77	1.13–3.18	**0.032**
Age^2^	0.996	0.992–0.999	**0.041**
Sex (female)	1.18	0.64–2.10	0.60
Randomization (SoC + CT)	3.18	1.80–5.92	**< 0.001**
Current smoking*	3.91	1.85–8.23	**< 0.001**
Stopped smoking*	1.91	1.03–3.63	**0.043**

This table shows the results of the multivariate logistic regression model evaluating the influence of age, sex, smoking status, and group allocation on the odds of having an incidental finding

*When compared to “never smoked”

**Fig. 3:**
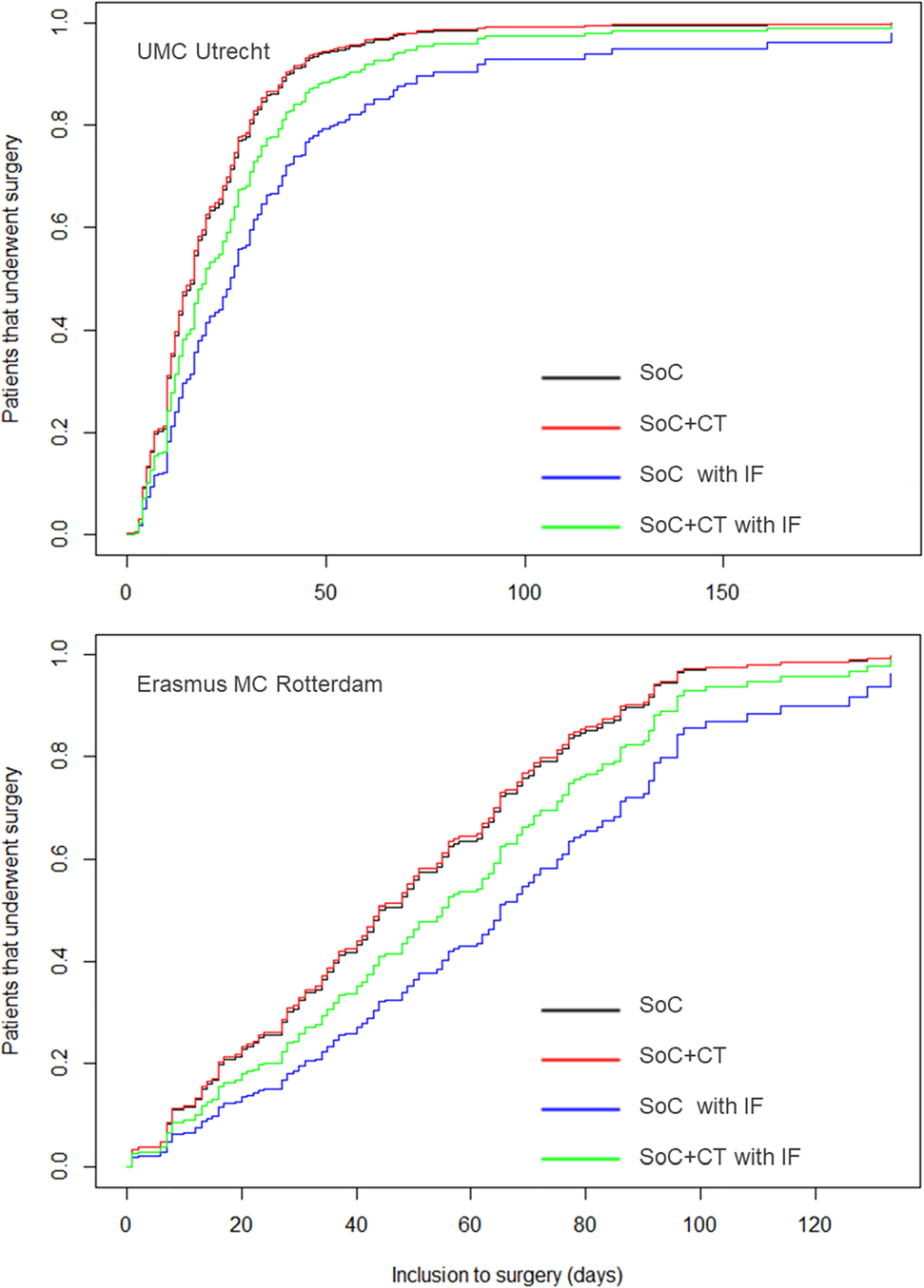
Time from inclusion to surgery for both groups, with and without incidental findings. IF, Incidental finding

The median time between inclusion and surgery was 21 days (17 days for 421 patients in the UMC Utrecht, 45 days for 197 patients in the Erasmus MC Rotterdam). The influence of routine CT and the presence of incidental findings are shown in Table 4 and Fig. 3. In the univariate analysis, not accounting for the presence or absence of incidental findings, there was no difference in time to surgery between the SoC and SoC+CT groups (hazard ratio (HR) 1.01, *p* = 0.90). The presence of an incidental finding significantly delayed the time to surgery (HR 0.56, *p* = 0.047), regardless of group allocation (HR for patients with an incidental finding in the SoC+CT group relative to the SoC group: 1.34, *p* = 0.42).

**Table 4:** Influence of randomization and presence of incidental findings on timing of surgery

Variable	Univariate hazard ratio	95% CI	*p*-value	Multivariate hazard ratio	95% CI	*p*-value
SoC + CT (relative to SoC)	1.01	0.86–1.18	0.90	1.02	0.87–1.21	0.79
Incidental finding	0.70	0.52–0.94	0.01	0.56	0.30–1.05	0.047
Incidental finding in SoC+CT				1.34	0.65–2.75	0.42

Cox regression stratified on center (UMCU and EMC), both univariate and multivariate including an interaction term between randomization and an incidental finding (*p* - values based on a likelihood ratio test). The hazard is interpreted as the instantaneous rate of undergoing surgery, with a ratio <1 indicating relative delay

## Discussion

When compared to a standard of care workup including a chest radiograph, the addition of routine preoperative noncontrast CT increased the rate of incidental findings by a factor of 3. In both groups, clinically relevant incidental findings were identified, such as lung cancer (in 0.5%), cardiac myxoma, sarcoidosis, or pneumonia. The largest difference with the addition of routine CT was observed in the category of pulmonary findings that required follow-up after surgery. Although routine CT did not delay the timing of surgery, the presence of incidental findings was associated with a longer time to surgery both in the SoC+CT and the SoC groups.

A routine preoperative CXR rarely leads to changes in surgical approach [11]. There are several indications to perform a preoperative CT in patients scheduled for cardiac surgery [1]. The main advantages of CT are that it provides detailed anatomical information in three dimensions, that it is non-invasive, and that image acquisition is less time-consuming compared to other modalities. With contemporary CT-scanners, preoperative scans can be performed with less than 1 mSv, limiting the consequences of radiation exposure [2]. In a large retrospective study, the use of chest radiography resulted in relevant incidental findings in only about 1.5% of patients [11]. In our study, relevant incidental findings were detected in a relatively comparable rate of 3.7% of patients in the SoC-group, with the slightly higher rate possibly explained by a higher detection rate in a prospective study. In contrast to CXR, relevant incidental findings are most prevalent in chest CT when compared to other imaging modalities and regions, owing to the detailed information and the many closely related organs [6]. Previous studies on incidental findings in cardiac surgery mentioned this as an additional reason to use preoperative CT [8, 9]. However, it could also be argued that these findings lead to unnecessary medical care and additional costs and potentially delay the timing of surgery [12].

Studies evaluating incidental findings prior to transcatheter valve procedures, where routine CT has been implemented on a large scale, observed a delay in timing of the intervention in only few patients [13, 14]. Similarly, we have found that the timing of surgery in both groups was only delayed by the presence of an incidental finding. Routine CT in a univariate analysis did not delay surgery, despite a higher rate of incidental findings. This is likely explained by the fact that most of these incidental findings only require follow-up after surgery, whereas many incidental findings on a chest radiograph need confirmation on CT prior to surgery. Although in part dependent on logistics and local waiting lists, our findings suggest the following with regard to daily practice. The smaller chance of an incidental findings on CXR is cancelled out by the larger delay caused by the inherent need of confirmation of the finding on CT. For this reason, the chances of surgical delay do not increase when implementing routine CT.

Two previous studies reported relevant incidental findings on preoperative contrast-enhanced CT in up to 50% of patients undergoing coronary artery bypass grafting, although this included aortic atherosclerosis, observed in 12–15% of patients [8, 9]. A lower rate of incidental findings of 18.7% was reported in patients undergoing various types of cardiac surgery [10]. The lower rate of incidental findings in our study has several explanations. Our definition was based on the need for any subsequent management, thereby excluding other minor incidental findings. Also, all of the studies mentioned, used contrast-enhanced imaging and included abdominal CT. Finally, these study samples were selected based on the availability of a preoperative scan, potentially selecting patients with a higher cardiovascular risk profile. Although the mean age in these studies was comparable to the mean age in our study, higher rates of risk factors such as smoking might have been present.

The question remains whether these incidental findings are risk-reducing or harmful for the patient. In our study, management of incidental findings classified as treatment or change of approach could be regarded as a benefit to the patient, as the finding prompted a change in care. Findings classified as non-invasive or invasive evaluation could be regarded as a burden to the patient, as these were findings that either did not lead to a specific diagnosis at the time of discharge (thus needing additional evaluation) or findings in which the additional evaluation ruled out clinically important pathology. Longer clinical follow-up could further evaluate to what extent patients were unnecessarily exposed to additional medical care, and how many findings led to clinically meaningful diagnosis after follow-up. The Fleischner guidelines on management of pulmonary nodules, which comprised most of the additional incidental findings in the SoC+CT group, have used a 1% risk of malignancy as a cut-off for follow-up [15]. In most cases, no growth is observed after one or two additional scans and malignancy can be ruled out. Only in about 2% of patients an invasive diagnostic test becomes necessary during follow-up, leading to the diagnosis of a lung cancer in approximately half of them [16]. The burden of additional tests with routine preoperative CT thus consists mainly of additional non-invasive follow-up.

The presence of a lung cancer in four patients (0.5%) in our study highlights the resemblance of part of our study population with that of lung cancer screening studies. Similar to the NELSON-study, the majority of our patients were male, with an average age of 66 years (NELSON study 59 years) and over half of our patients had a history of smoking [17]. The first screening round in the NELSON study revealed a lung cancer in 0.9% of patients. Our study’s estimated prevalence should be regarded with the appropriate degree of uncertainty, given the low number of events. However, our findings suggest that routine preoperative CT might have an additional benefit in patients with a history of smoking undergoing cardiac surgery, specifically those patients with a high pretest risk of lung cancer. Adjusted for age, sex, and randomization, we observed a higher rate of incidental finding, with odds that were two (in patients who stopped smoking) to four (in active smokers) times as high as in patients who never smoked. This finding has previously been reported in patients with stable angina undergoing cardiac CT [7]. However, compared to such a population, patients scheduled for cardiac surgery might have additional benefit from significant incidental findings. Not only does it allow for (oncological) treatment, but it also allows for subsequent adjustment or cancellation of the cardiac surgical procedure. This way, unnecessary surgery can be avoided in patients with a shorter life expectancy, or cardiac rehabilitation does not interfere in patients requiring urgent chemotherapy, radiation, or lung surgery.

There are several limitations in our study that need to be taken into account. Our definition of incidental findings was based on the presence of subsequent management. This means that minor incidental findings, e.g., arthritis and hepatic steatosis, were not captured. Also, this definition is specific to patients being prepared for cardiac surgery. For instance, because a lung function test is performed in all patients at risk for pulmonary disease, the presence of emphysema on preoperative CT did not trigger additional management and was not included as an incidental finding. With regard to clinically relevant incidental findings such as aortic dilatation or lung cancer, our study was not powered to evaluate the diagnostic yield of a workup with or without routine CT. Finally, the lack of follow-up after discharge prohibits an interpretation of the clinical consequences of the findings that required clinical follow-up.

In patients scheduled for cardiac surgery, the addition of routine CT-screening will increase the prevalence of incidental findings, mainly by increasing the rate of pulmonary findings requiring follow-up after surgery. Clinically important findings necessitating treatment or change of surgery can be found in approximately 1–2% of patients, both with and without CT. Incidental findings are more prevalent in patients with a history of smoking, and preoperative CT might increase the yield of identifying lung cancer in these patients. Routine CT-screening does not delay the timing of surgery, although the presence of incidental findings does prolong this interval.

## Supplementary Information

ESM 1 (DOCX 336 kb)

## Abbreviations

CXR: Chest radiograph
SoC: Standard of care

## Declarations

### Guarantor

The scientific guarantor of this publication is Ricardo P.J. Budde.

### Statistics and biometry

No complex statistical methods were necessary for this paper.

### Study subjects or cohorts overlap

Since this is a sub-analysis, the study subjects have been previously reported in the main paper, but no overlap is present in the reported outcomes.

### Methodology

• prospective

• randomized controlled trial

• multicenter study
