# Supplementary material for: Incidental findings on routine preoperative noncontrast chest computed tomography and chest radiography prior to cardiac surgery in the multicenter randomized controlled CRICKET study
Source: Eur Radiol. 2022 Jul 19;33(1):294–301. doi: 10.1007/s00330-022-09001-0 (PMC9755074; doi:10.1007/s00330-022-09001-0)
Supplement: Supplementary file 1 — (DOCX 336 kb) [file 330_2022_9001_MOESM1_ESM.docx]

**Supplementary data**

Supplementary data to: Incidental findings on routine preoperative noncontrast chest computed tomography and chest radiography prior to cardiac surgery in the multicenter randomized controlled CRICKET study; W.G. Knol, A.M. den Harder, L.M. de Heer et al

Contents

[Table S1 Type of surgery planned 2](#_Toc101443958)

[Table S2 Diagnosis and Management of patients in the SoC group 3](#_Toc101443959)

[Table S3 Diagnosis and management of patients in the SoC+CT group 4](#_Toc101443960)

# Table S1 Type of surgery planned

| Type of surgery | SoC group | SoC+CT group |
| --- | --- | --- |
| Isolated CABG | 39.5% (171/433) | 37.1% (159/429) |
| Isolated AVR^a^ | 21.0% (91/433) | 25.4% (109/429) |
| AVR+CABG | 11.3% (49/433) | 8.6% (37/429) |
| Mitral valve surgery^a^ | 17.6% (76/433) | 20.7% (89/429) |
| Other | 10.6% (46/433) | 7.9% (34/429) |
| AVR + additional procedure (aneurysmectomy, ablation surgery, tricuspid valve surgery) | 2.3% (10/433) | 3.0% (13/429) |
| CABG + additional procedure (aneurysmectomy, ablation surgery, septal defect) | 2.1% (9/433) | 1.2% (5/429) |
| Aortic (root) surgery | 1.6% (7/433) | 1.2% (5/429) |
| Tricuspid valve surgery | 0.9% (4/433) | 0.5% (2/429) |
| Septal defect^a^ | 1.2% (5/433) | 0.2% (1/429) |
| LVAD implantation | 0.2% (1/433) | 0.7% (3/429) |
| Myectomy (Morrow) | 0.9% (4/433) | 0.5% (2/429) |
| Pulmonary valve surgery | 0.5% (2/433) | 0.7% (3/429) |
| Left ventricular aneurysmectomy | 0.5% (2/433) | 0 |
| Excision of myxoma | 0.5% (2/433) | 0 |
| Closure of coronary fistula | 0 | 0.2% (1/429) |

No significant differences were present between groups after randomization. Proportions are given as % (n).

a) a minimally invasive surgical approach was used in two patients undergoing mitral valve surgery, a patient undergoing closure of a septal defect and a patient undergoing aortic valve replacement.

AVR = Aortic valve replacement, CABG = Coronary artery bypass grafting, LVAD = Left ventricular assist device.

# Table S2 Diagnosis and Management of patients in the SoC group

| **SoC (no of patients)** | **Diagnosis** | **Management Classification** | **Type of management required** | |
| --- | --- | --- | --- | --- |
|  |  |  | **Direct** | **Follow-up** |
| 1 | Cardiac myxoma | Treatment | 1 | 0 |
| 2 | Lung cancer | Treatment | 2 | 0 |
| 1 | Abdominal mass, no malignancy | Invasive evaluation | 1 | 0 |
| 1 | Pulmonary nodule | Invasive evaluation | 1 | 0 |
| 1 | Pulmonary parenchymal abnormalities | Invasive evaluation | 1 | 0 |
| 1 | Pulmonary nodule | Non-invasive evaluation | 0 | 1 |
| 3 | Aortic dilatation | Non-invasive evaluation | 0 | 3 |
| 5 | Suspected pulmonary nodule, ruled out by CT | CT | 0 | 5 |
| 1 | Pulmonary parenchymal abnormalities | CT | 0 | 1 |

# Table S3 Diagnosis and management of patients in the SoC+CT group

| **SoC+CT (no of patients)** | **Diagnosis** | **Management Classification** | **Type of management required** | |
| --- | --- | --- | --- | --- |
|  |  |  | **Direct** | **Follow-up** |
| 2 | Lung cancer | Treatment | 2 | 0 |
| 1 | Sarcoidosis | Treatment | 1 | 0 |
| 2 | Aortic dilatation | Changed approach | 2 | 0 |
| 1 | Liver cirrhosis | Changed approach | 1 | 0 |
| 1 | Pneumonia | Changed approach | 1 | 0 |
| 3 * | Mass, no malignancy | Invasive evaluation | 3 | 0 |
| 18 | Pulmonary nodule | Non-invasive evaluation | 0 | 18 |
| 5 | Groundglass lesion | Non-invasive evaluation | 0 | 5 |
| 3 # | Mass, no malignancy | Non-invasive evaluation | 2 | 1 |
| 3 | Pulmonary parenchymal abnormalities | Non-invasive evaluation | 1 | 2 |
| 2 | Pulmonary fibrosis | Non-invasive evaluation | 2 | 0 |
| 2 | Adrenal adenoma | Non-invasive evaluation | 0 | 2 |
| 2 | Mediastinal lymphadenopathy | Non-invasive evaluation | 0 | 2 |
| 1 | Left ventricular intramural thrombus | Non-invasive evaluation | 1 | 0 |
| 1 | Liver mass | Non-invasive evaluation | 0 | 1 |
| 1 | Renal dilatation | Non-invasive evaluation | 0 | 1 |
| 1 | Thyroid calcified nodules | Non-invasive evaluation | 0 | 1 |

* Masses were located in the breast, adrenal gland and paracardial.

# Masses were located in the pancreas, kidney and pleura.

**Figure S1**


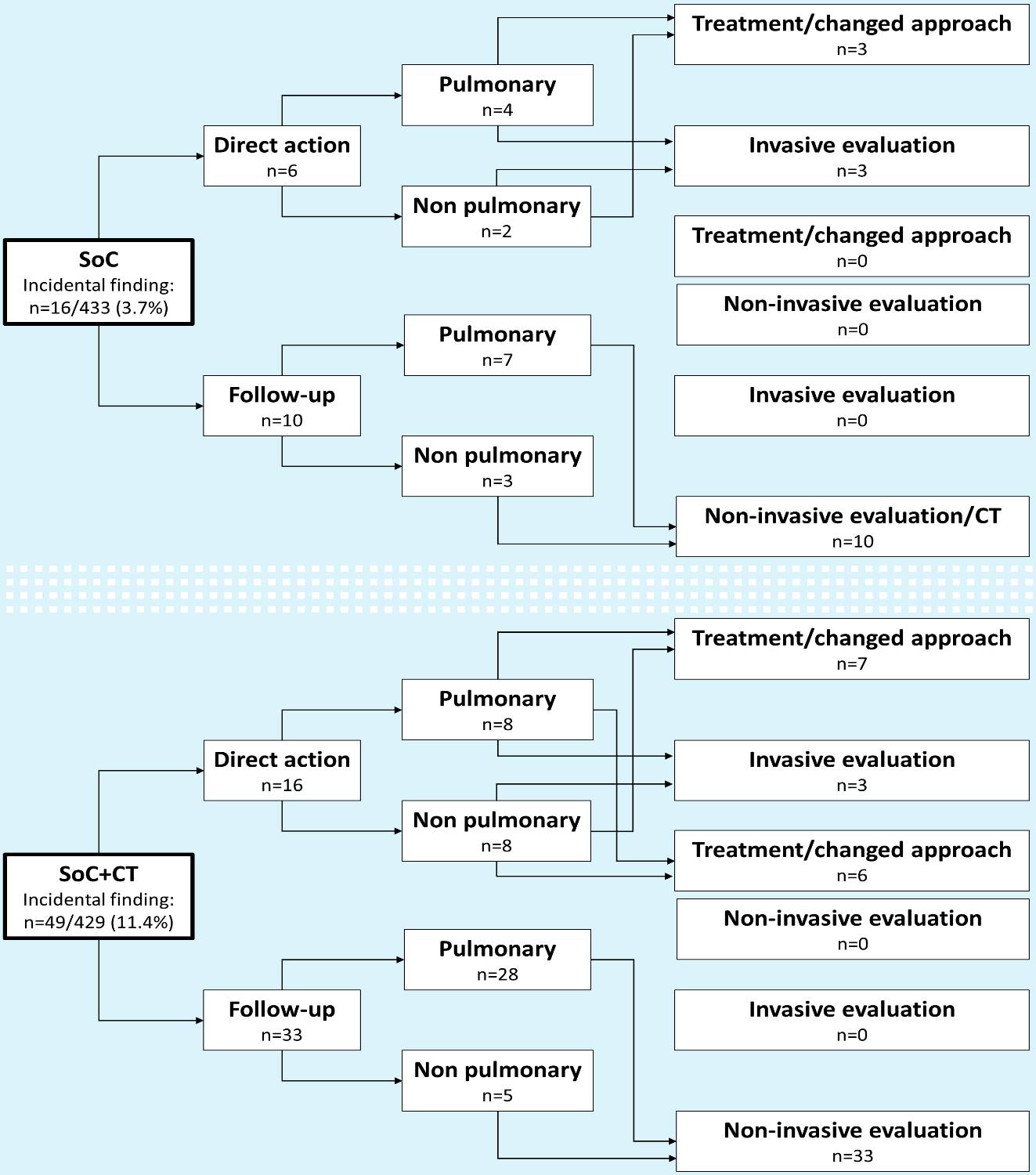


A flowchart of the incidental findings in both groups (SoC = Standard of Care, SoC+CT = Standard of Care with routine CT-scan). The number of patients categorized as direct findings or follow-up are given, subcategorized as pulmonary or non-pulmonary findings. To the right, the management is given for both groups.
